# Transcriptome Analyses Revealed the Genetic Advantages in Polygynous Males of *Tylonycteris pachypus*

**DOI:** 10.1002/ece3.72116

**Published:** 2025-09-10

**Authors:** Chuyu Lv, Libiao Zhang, Ke Li, Panyu Hua

**Affiliations:** ^1^ School of Ecological and Environmental Sciences East China Normal University Shanghai China; ^2^ Institute of Zoology Guangdong Academy of Sciences Guangzhou China

**Keywords:** *de novo* assembly, polygynous group, RNA‐seq, sexual selection, *Tylonycteris pachypus*

## Abstract

Sexual selection is widely recognized as a key driver of evolutionary processes; However, research on this topic in bats remains limited despite their considerable ecological importance. The flat‐headed bat *Tylonycteris pachypus* , a small bamboo‐roosting species, typically forms either polygynous groups (one male with multiple females) or male‐only groups. Previous studies have shown that males roosting with females experience significantly higher reproductive success, despite the absence of documented morphological sexual dimorphism. In this study, we constructed a reference transcriptome for *T. pachypus* using RNA‐seq, combining *de novo* assembly and transcript‐guided assembly based on a closely related species. We compared gene expression profiles of brain tissue between solitary males and polygynous males from the Chongzuo region in Guangxi, China. A total of 107 differentially expressed genes (DEGs) were identified, including *SEMA6A*, *KCTD12*, *CLTC* and *ALDH1L1*, etc., upregulated in polygynous males, while *VANGL2*, COTL1 and *IL10RB* were upregulated in solitary males. These results suggest that polygynous males may possess superior survival and reproductive potential compared to solitary males. KEGG enrichment analysis revealed that polygynous males were significantly enriched in pathways including “lysosome” and “endocrine and other factor‐regulated calcium reabsorption”. GO enrichment analysis also indicated various differences between the two male groups. Our results demonstrate distinct gene expression patterns between the two male groups, offering preliminary molecular evidence for adaptive evolution driven by sexual selection in *T. pachypus* .

## Graphical abstract

This study explores the role of sexual selection in Tylonycteris pachypus, with a particular focus on gene expression differences between solitary and polygynous males. RNA‐seq analysis identified 107 differentially expressed genes, with polygynous males exhibiting upregulation of genes associated with survival and individual health. The results offer molecular insights into the potential mechanisms underlying sexual selection and adaptive evolution in T. pachypus, highlighting key pathways that may contribute to male reproductive success.

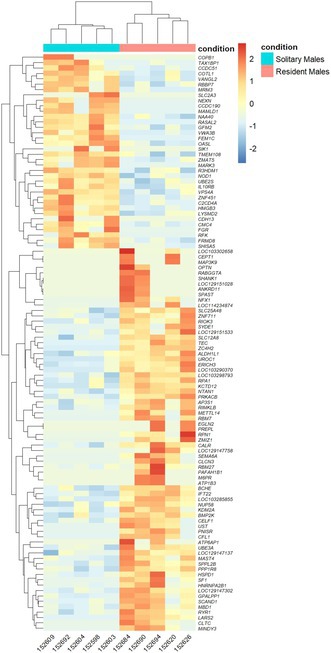

## 1 Introduction

Sexual selection, first proposed by Darwin, explains the persistence of traits that confer no apparent advantage under natural selection. It is typically divided into two categories: intersexual selection and intrasexual competition. Sexual selection drives the evolution of morphological and behavioral traits by enhancing reproductive success. A classic example is the elaborate plumage of peacock tails, which is positively correlated with male reproductive success (Petrie et al. 1991). Although such traits may impose survival costs, they provide substantial reproductive benefits. Moreover, sexual selection plays a crucial role in shaping population responses to environmental changes (Candolin and Heuschele 2008). Shifts in environmental conditions can alter the intensity and targets of sexual selection, leading to the amplification of specific genetic traits and influencing species‐wide adaptability. As a distinct component of natural selection, sexual selection is a key driver of evolutionary processes with significant ecological implications.

Bats (order Chiroptera) are the only mammals capable of powered flight. They are classified into two primary suborders: Yinpterochiroptera and Yangochiroptera. Bats are both highly diverse and widely distributed, playing critical roles in maintaining ecological balance. They contribute to pest control (Wanger et al. 2014), plant pollination, and seed dispersal, all of which are essential for sustaining ecosystem diversity. The absence of significant differences in body size or skeletal features between male and female bats has long supported the assumption of minimal sexual dimorphism in this group (Lu et al. 2014). Recent studies suggest that sexual selection in bats may primarily act on traits associated with soft tissues, epidermal glands, or related structures (Muñoz‐Romo et al. 2021). Despite these findings, research on such traits remains limited and fragmented.

The flat‐headed bat ( *Tylonycteris pachypus* : Chiroptera, Vespertilionidae) is distributed across South and Southeast Asia, including Guangxi, China (Zhang et al. 2002). It roosts in small bamboo stems, breeds once a year, and typically produces twins (Medway and Marshall 1970). Research on *T. pachypus* has provided valuable insights into its behavioral, ecological, and physiological traits (Zhang et al. 2002, 2013), while its sexual selection mechanisms remain poorly understood, with no reported evidence of sexual dimorphism. Our previous study on the dispersal behavior of *T. pachypus* showed that offspring of both sexes typically exhibit localized dispersal within the same bamboo forest and rarely migrated to other forests, highlighting their strong habitat fidelity (Zhang et al. 2013). *T. pachypus* typically forms either polygynous groups or male‐only groups. Resident males are those that roost in stable, polygynous groups with one or more females, while solitary males are those observed in male‐only aggregations. Occasionally, individuals in roosting groups switch among adjacent internodes (Medway 1972; Zhang et al. 2004). However, once this switching occurs, males roosting with females continue to roost with the same individuals, and solitary males remain solitary, suggesting that the group composition is generally stable (Zhang et al. 2004). According to our previous study on the parentage of *T. pachypus* , resident males produce more offspring than other males and exhibit a stronger tendency to monopolize paternity within litters (Hua et al. 2011). Given the absence of observable morphological differences between resident and solitary males, molecular approaches such as transcriptome sequencing represent the most viable strategy to investigate sexual selection mechanisms in *T. pachypus* .

RNA‐seq, a key technology of next‐generation sequencing, provides comprehensive insights into RNA sequences, structures, and expression levels (Gao et al. 2018). Since its introduction, it has become a fundamental tool in molecular biology, widely used for gene function studies, transcriptome profiling, and differential gene expression analysis (He et al. 2019; Ye et al. 2018), and it was particularly valuable for research on non‐model species (Xu et al. 2013).

Resident and solitary males exhibit no phenotypic differences but differ in clustering behavior and reproductive success, providing a unique opportunity to explore the factors influencing sexual selection in *T. pachypus* . Given the brain's central role in hormone metabolism, neuronal function, and stress regulation, we selected brain tissue for transcriptomic analysis. As a key organ in mediating responses to social and reproductive behaviors, the brain may exhibit sexual dimorphism that precedes gonadal differentiation (Dewing et al. 2003). By focusing on brain tissue, we aim to uncover potential physiological and genetic differences that could underlie reproductive success, even in the absence of visible sexual dimorphism. To investigate the genetic differences, we constructed a reference transcriptome of *T. pachypus* using RNA‐seq. We hypothesize that transcriptomic differences between resident and solitary males may reveal key factors driving sexual selection. Specifically, we aim to test the following hypotheses: (i) Gene expression profiles and functional enrichment will significantly differ between males from the two groups. (ii) Resident males, with more stable social structures and higher reproductive success (Hua et al. 2011), will show differential expression of genes potentially associated with reproduction, sociality, or health.

## 2 Materials and Methods

### 2.1 Sampling, RNA Extraction and Sequencing

This study was carried out in strict accordance with the guidelines of Regulations for the Administration of Laboratory Animals (Decree No. 2 of the State Science and Technology Commission of the People's Republic of China on November 14, 1988). We obtained approval for this study from the Guangdong Entomological Institute Administrative Panel on Laboratory Animal Care. Permission from the landowners was also obtained. Samples of *Tylonycteris pachypus* were collected from the Gaoxiang Village, Chongzuo region of Guangxi province, China. The village contains multiple bamboo forests, each consisting of dozens to over a hundred bamboo stems. These bamboo forests are located in close proximity to each other, with minimal distance between them. We carried out sampling across different bamboo forests in the area, ensuring a broad representation of the local bat population. A total of 15 healthy adult individuals (5 resident males, 5 solitary males and 5 females) were collected, with species identification verified by qualified personnel. Sample collection was completed within 2 days in August. Prior to dissection, the bats were euthanized using the cervical dislocation method, which is commonly used for small mammals. The procedure was performed by applying pressure to the bat's head with the thumb and index finger to prevent biting, while the hind leg was grasped and pulled quickly to dislocate the vertebrae, resulting in immediate death. This method is designed to end the bat's life quickly and minimize suffering. All specimens were immediately dissected upon collection. The tissues obtained from dissection were rapidly frozen in liquid nitrogen and then stored at −80°C for further use.

Total RNA was extracted using TRIzol Reagent (Invitrogen, USA), following the manufacturer's protocol (Chomczynski and Sacchi 1987). RNA quality was rigorously assessed, including purity measurement (NanoDrop spectrophotometer) and integrity evaluation (Agilent 2100 Bioanalyzer). Only high‐quality samples (RIN ≥ 7, OD260/280 ratio 1.8–2.0) were used for subsequent analyses (Schroeder et al. 2006). PolyA enrichment was performed using Oligo dT primers to capture mRNA and form cDNA, ensuring the removal of rRNA. After testing the quality of the libraries, each library was sequenced individually on the Illumina HiSeq 2500 platform, and 150 bp paired‐end reads were generated at OE Biotech Co. Ltd., Shanghai, China.

### 2.2 Transcriptome Assembly Approaches for *Tylonycteris pachypus*

**FIGURE 1:**
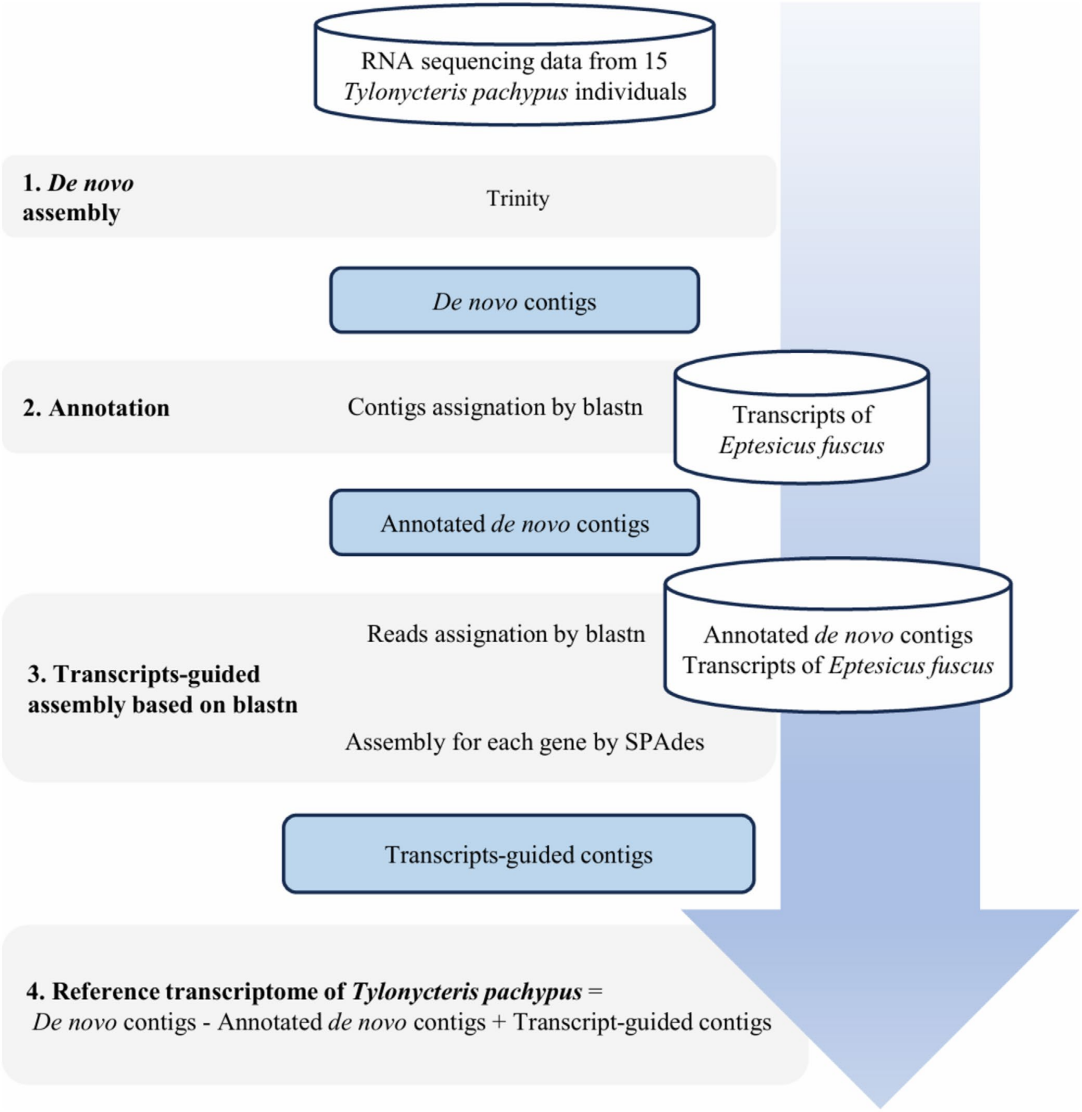
Overview of the transcriptome assembly workflow. Step 1 involves *de novo* assembly, while Steps 2 onward describe the transcript‐guided assembly process using *Eptesicus fuscus* transcripts. Step 4 illustrates the computation of the final reference transcriptome of *T. pachypus*.

All sequencing data from 15 individuals were incorporated into the construction of the *Tylonycteris pachypus* reference transcriptome to enhance its reliability and completeness. Before assembly, raw RNA‐seq data were subjected to quality control and preprocessing. Paired‐end reads were processed using Trimmomatic v0.36 to remove adapter sequences and low‐quality reads (Bolger et al. 2014). The transcriptome assembly workflow is shown in Figure 1. Since *Tylonycteris pachypus* lacks both a reference genome and a reference transcriptome, *de novo* assembly alone presented several challenges. To overcome the limitations, we followed Ungaro et al.'s (2017) method, which combines *de novo* assembly with guided assembly based on closely related species (Ungaro et al. 2017).

#### 2.2.1 *De Novo* Assembly

Lu compared the performance of various *de novo* assembly software and concluded that Trinity provides the highest assembly completeness and contiguity (Lu et al. 2013). In this study, transcriptome assembly was performed using Trinity (Friedman et al. 2011). The resulting contigs were clustered using CD‐HIT‐EST v4.7 with a sequence similarity threshold of 0.9 (Fu et al. 2012).

#### 2.2.2 Transcripts‐Guided Assembly

We selected *Eptesicus fuscus* (the big brown bat) as a closely related species and downloaded its RefSeq rna.fna transcripts file from the NCBI assembly GCF_027574615.1 for annotation. A database of *E. fuscus* transcripts was constructed using makeblastdb in BLAST (Altschul et al. 1990). All contigs from the *de novo* assembly were aligned to this database using BLASTn (qcov_hsp_perc = 70, *e*‐value = 1e‐5, word size = 9, perc_identity = 70, and max_target_seqs = 1). The best alignment for each contig was retained to annotate the *de novo* contigs with transcript information from *E. fuscus* , resulting in a set of annotated *de novo* contigs.

The transcript of *E. fuscus* was merged with the annotated *de novo* contigs to construct a local database. Quality‐controlled reads were aligned to this local database using BLASTn once more (*e*‐value = 1e‐5). Each gene could align to multiple reads, which were collected into gene‐specific folders named accordingly. SPAdes v3.15.4 (−careful, −cov‐cutoff auto, *k* = 21, 33, 55, 77, −rna) was used to assemble each contig individually (Prjibelski et al. 2020). All assembly outputs were further compared with *E. fuscus* transcripts using BLAST (*e*‐value = 1e‐5), and the longest contig from the hits was retained as the representative sequence for each gene. SPAdes, which supports hybrid assembly of long and short reads, was used instead of Trinity in this step to minimize bias introduced by relying on a single tool (Prjibelski et al. 2020). The resulting assemblies were combined to generate a set of transcript‐guided contigs.

The final reference transcriptome of *Tylonycteris pachypus* was generated by subtracting the annotated *de novo* contigs from the *de novo* contigs and then adding the transcripts‐guided contigs. The annotated *de novo* contigs were subtracted because the transcripts‐guided contigs were assembled based on them, resulting in overlapping regions between the two sets. The final transcriptome was evaluated using TransRate v1.0.3 for metrics such as N50 and GC content (Smith‐Unna et al. 2016), and transcriptome completeness was assessed using BUSCO v5.7.1 with the *laurasiatheria_odb10* database (Manni et al. 2021).

### 2.3 Measurement of Gene Expression

Quantification of transcriptomes of resident and solitary males was performed using RSEM v1.3.1 (Li and Dewey 2011). Indexes were constructed from the previously assembled final transcriptome using Bowtie2 v2.3.4.1. Expression levels of each gene across individual transcriptomes were then calculated using the rsem‐calculate‐expression function within RSEM (Langmead and Salzberg 2012).

### 2.4 Identification and Enrichment Analysis of Differentially Expressed Genes

The expected_counts values from RSEM were imported into DESeq2 v1.44.0 (Love et al. 2014) for normalization and subsequent identification of differentially expressed genes (DEGs). On this step, we focused on the subset of genes with functional annotations, and only these were retained for further interpretation. The DEGs were uploaded to the DAVID (Database for Annotation, Visualization, and Integrated Discovery) online platform (https://davidbioinformatics.nih.gov/home.jsp), where the Gene ID Conversion tool was used to convert the DEGs to unified Gene Symbols. The converted results were then uploaded to KOBAS (http://bioinfo.org/kobas) for KEGG and GO enrichment analyses. We adjusted the significant level by a correction for false discovery rate (FDR) at < 0.05 with Benjamini‐Hochberg correction method (Benjamini et al. 2001).

## 3 Results

### 3.1 Transcriptome Assembly of *Tylonycteris pachypus*

The raw transcriptome data generated from sequencing totaled approximately 289.14 GB. The *de novo* assembly yielded 740,782 contigs. Using *E. fuscus* transcripts for annotation, 40,215 contigs were obtained. The final reference transcriptome was constructed by integrating the *de novo* assembly and transcriptome‐guided assembly results, excluding redundant annotated contigs. Assembly evaluation metrics are presented in Table 1. The final transcriptome contained 716,849 contigs, with an N50 value of 1308 bp and an average contig length of approximately 823.81 bp. The GC content was 49.67%, showing no significant deviation. BUSCO analysis using the *laurasiatheria_odb10* database identified 74.2% of the expected conserved single‐copy orthologs as complete.

**TABLE 1:** Assembly statistics of reference transcriptome of *Tylonycteris pachypus* .

Metric	Value
Number of contigs	716,849
Mean contig length	823.81 bp
N50	1308 bp
GC content	49.67%
Complete BUSCOs	74.2%

### 3.2 Identification of Differentially Expressed Genes (DEGs)

**FIGURE 2:**
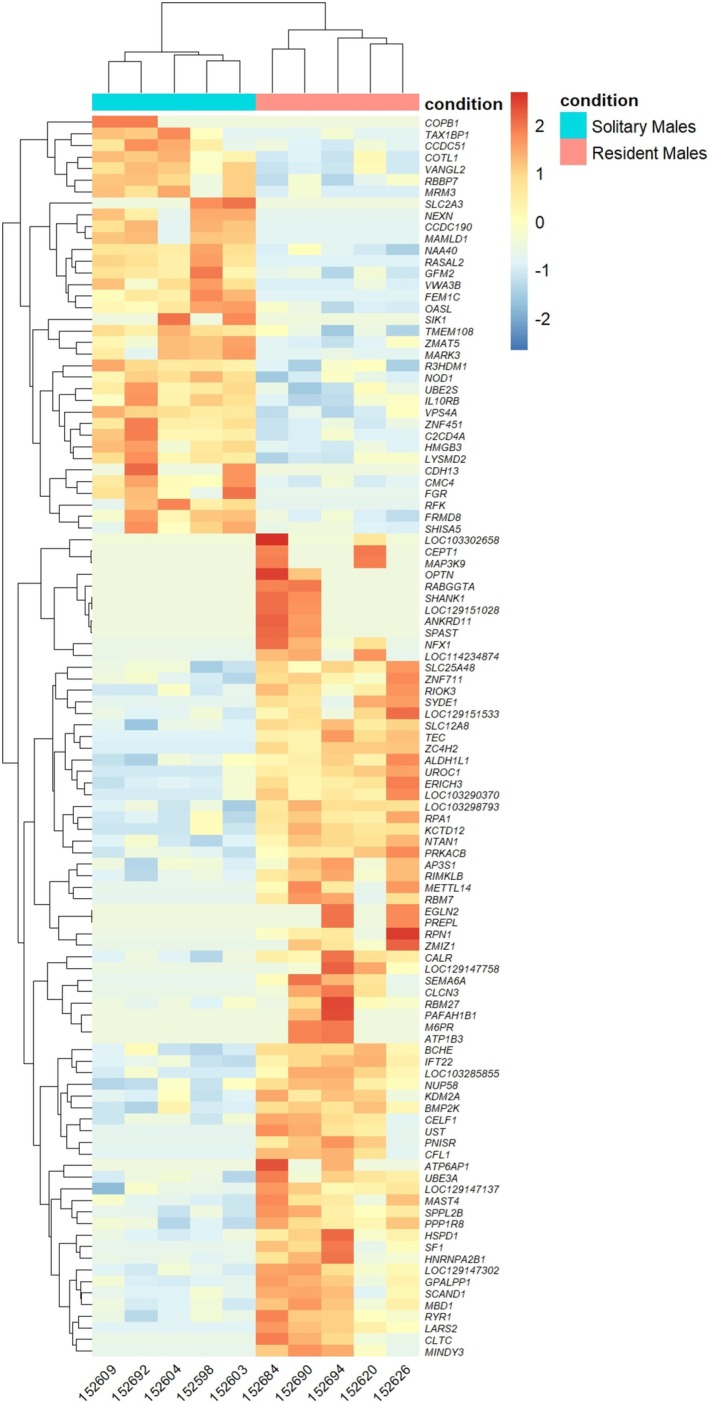
Heatmap of DEGs between resident and solitary male *Tylonycteris pachypus* . The heatmap illustrates the expression levels of DEGs between resident males and solitary males. Red indicates higher gene expression levels, while blue indicates lower levels. Gene clustering relationships are shown on the left, and sample IDs are listed below.

**FIGURE 3:**
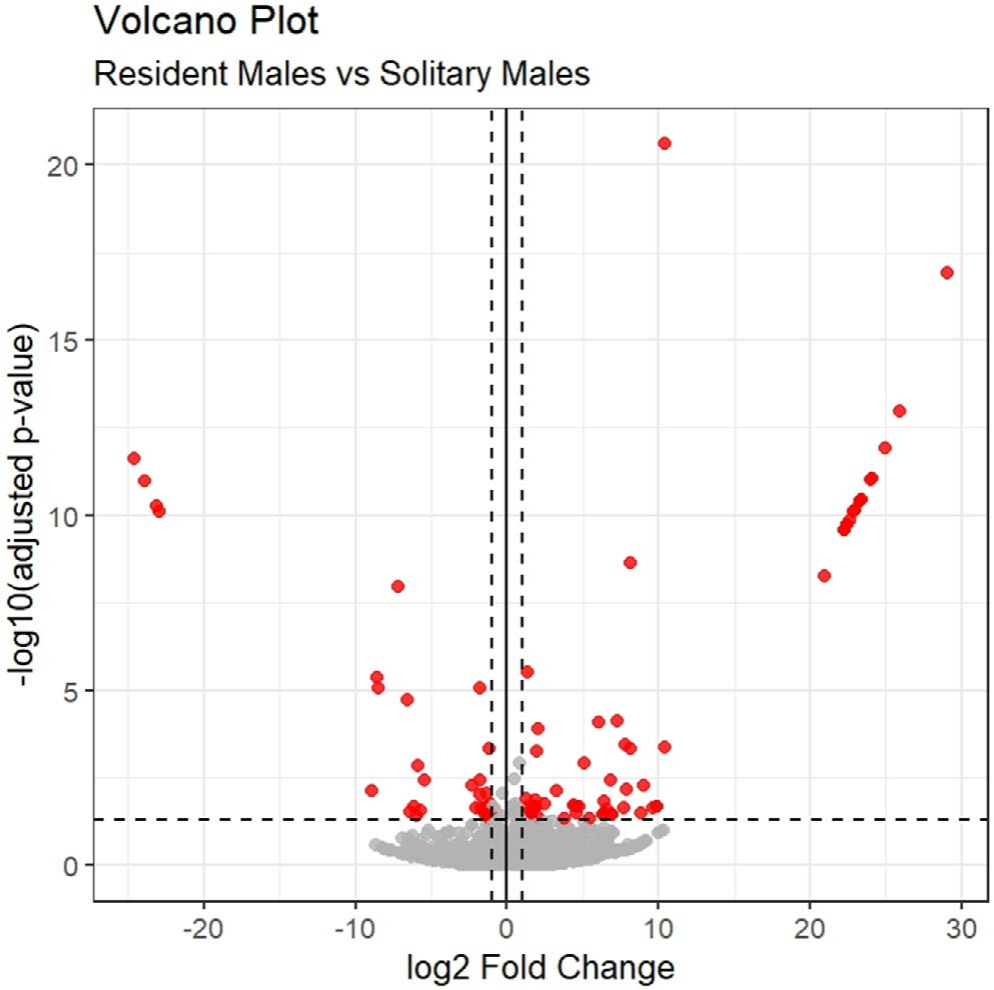
Volcano plot of DEGs between resident and solitary male *Tylonycteris pachypus* . The volcano plot depicts 40,215 genes, with the x‐axis representing the log2 fold change (Log2 FC) and the y‐axis representing −log10 P. Red points indicate DEGs that meet both *p*‐value and fold change thresholds.

A total of 107 DEGs were identified in this study (FDR < 0.05). Of these, 71 genes were significantly upregulated in resident males compared to solitary males, while 36 genes were significantly downregulated. As shown in Figure 2, the gene expression patterns of resident and solitary males exhibit clear separation, suggesting that the DEGs between the two groups are involved in multiple biological processes. The observation supports the classification of resident and solitary males based on differences in mate acquisition and offspring production. Figure 3 illustrates the overall distribution of DEGs, with many significant DEGs (red points) meeting the thresholds for statistical significance (*p*‐value) and fold change (log2 fold change).

### 3.3 Enrichment Analysis

**FIGURE 4:**
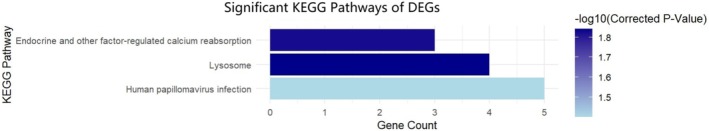
Significantly enriched KEGG pathways of DEGs. The figure illustrates the KEGG pathway enrichment analysis based on DEGs. The y‐axis lists the significantly enriched KEGG pathways, while the x‐axis represents the number of enriched genes (Gene Count) in each pathway. Bar length indicates the number of enriched genes, and the color gradient reflects the corrected significance level (−log10 Corrected *p*‐value), with darker colors representing higher significance.

**FIGURE 5:**
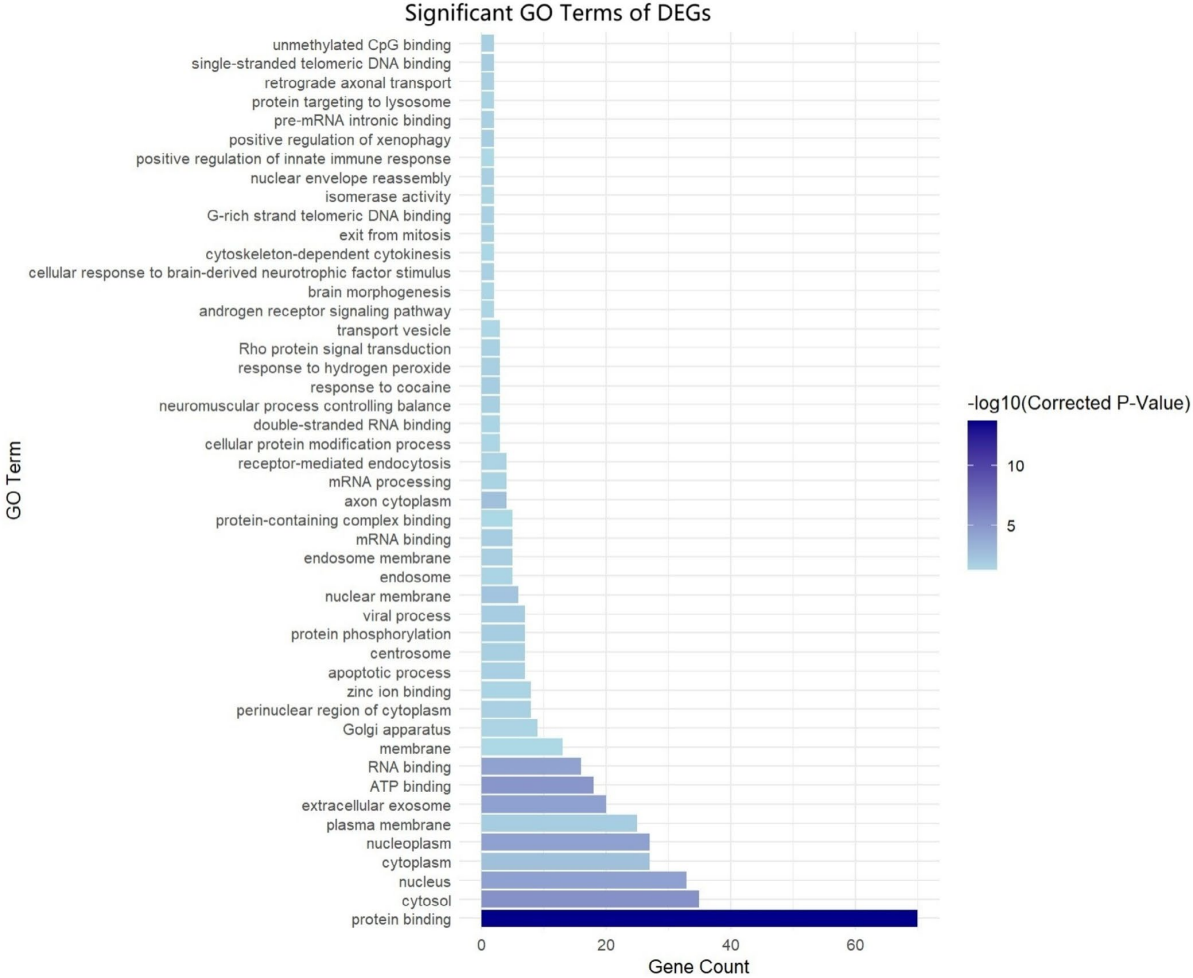
Significantly enriched GO terms of DEGs. The figure shows the significantly enriched Gene Ontology (GO) terms based on DEGs. The y‐axis lists the enriched GO terms, while the x‐axis represents the number of enriched genes (Gene Count) for each term. The color gradient indicates the corrected significance level (−log10 Corrected *p*‐value), with darker colors denoting higher significance.

To further elucidate the functions of differentially expressed genes (DEGs), enrichment analysis was performed using the KOBAS online platform. In the KEGG database, three pathways were significantly enriched (Figure 4): Endocrine and other factor‐regulated calcium reabsorption, Lysosome, and Human papillomavirus infection. In the GO database, a total of 47 categories were significantly enriched (Figure 5). Among these, Protein binding had the highest number of enriched genes and showed the most significant enrichment. Other terms, such as Nucleus and Cytosol, also exhibited substantial gene enrichment.

## 4 Discussion

### 4.1 DEGs Highlighting the Advantages of Resident Males

Our transcriptomic analysis revealed significant gene expression differences between resident and solitary males of *Tylonycteris pachypus* , providing insights into the molecular mechanisms underlying their ecological and behavioral divergence.

In the brains of resident males, genes such as *SEMA6A*, *CLTC*, *KCTD12*, *CFL1*, *RYR1*, and *CLCN3* exhibited significant upregulation, suggesting enhanced adaptations in synaptic plasticity, neuronal signaling, and social interactions. Specifically, elevated expression of *SEMA6A*, an axon guidance molecule, could enhance coping mechanisms in complex social contexts by facilitating information transfer and social memory formation (Carulli et al. 2021; Rünker et al. 2011). Increased levels of *CLTC*, encoding the clathrin heavy chain essential for synaptic vesicle endocytosis and recycling (Manti et al. 2019), likely promote efficient neuronal signaling and sustained information flow during intricate social interactions. Additionally, *KCTD12*, an auxiliary regulator of GABA_B receptors involved in inhibitory neurotransmission (Teng et al. 2019), may finely modulate neuronal excitability and emotional responses, supporting emotional stability and rapid behavioral responsiveness (Cathomas et al. 2015). *CFL1* (Cofilin 1), an actin‐depolymerizing protein crucial for neuronal morphology and synaptic plasticity (Hotulainen et al. 2005), potentially enhances neuronal adaptability to rapid environmental changes, improving cognitive flexibility (Namme et al. 2021). *RYR1* and *CLCN3* regulate intracellular calcium and chloride ion fluxes, respectively, essential for synaptic transmission and ionic homeostasis. Upregulated *RYR1* expression ensures precise calcium dynamics, promoting accurate neuronal signaling and synaptic stability (Lanner et al. 2010; Verkhratsky 2006). Similarly, enhanced *CLCN3* expression stabilizes neuronal membrane potentials (Stauber et al. 2012), optimizing neural network function under intense social pressure. Collectively, these gene expression alterations likely underpin neurological adaptations enabling resident males to excel in prolonged social interactions, group cohesion, and effective communication.

Interestingly, genes associated with neural regulation, including *VANGL2* and *COTL1*, exhibited significant upregulation in solitary males. *VANGL2*, involved in regulating cell polarity through the Wnt/PCP signaling pathway, is essential for neuronal migration, axon guidance, and synaptic localization (Koehl et al. 2022). Its enhanced expression may increase neuronal polarity plasticity, facilitating occasional social interactions. Similarly, *COTL1* regulates actin dynamics critical for neuronal morphology and synaptic remodeling (Li et al. 2018) enabling rapid behavioral adjustments to dynamic environments. Thus, gene expression profiles in solitary males appear tailored to transient rather than sustained social interactions. These neurological adaptations likely support alternative reproductive strategies, such as sneak mating, enabling reproductive success without direct competition with dominant resident males, aligning with previous research findings.

Furthermore, distinct differences in energy management strategies were noted between resident and solitary males. Resident males showed significant upregulation of genes including *HSPD1*, *ALDH1L1*, *SLC25A48*, and *LARS2*, indicating diverse and efficient energy utilization mechanisms. Specifically, *HSPD1* functions as a molecular chaperone aiding cellular resilience to environmental and physiological stresses (Hu et al. 2022). *ALDH1L1*, abundantly expressed in astrocytes, sustains central nervous system energy metabolism and neuronal homeostasis (Yang et al. 2011). Enhanced expression of *SLC25A48* likely augments mitochondrial fatty acid metabolism, providing stable and sustained energy availability. *LARS2* further promotes mitochondrial protein synthesis and metabolic efficiency, bolstering overall energy utilization (Pierce et al. 2013). Conversely, solitary males exhibited increased expression of *RFK* and *GFM2*, primarily supporting basal metabolic requirements (Balasubramaniam and Yaplito‐Lee 2020; Smits et al. 2010), indicative of conservative energy optimization.

Regarding immune and inflammatory responses, no significant upregulation of classic inflammatory factors was observed in resident males. However, solitary males displayed widespread upregulation of immune or inflammatory signaling genes, such as *IL10RB*, *NOD1*, *OASL*, *TAX1BP1*, and *FGR*, suggesting chronic or excessive immune activation. Prolonged inflammation or immune stress typically impairs neural plasticity and consumes substantial energy, potentially hindering competitive and reproductive capabilities. *IL10RB* is a critical component of the interleukin‐10 (IL‐10) receptor complex and mediates IL‐10's anti‐inflammatory signaling. It is expressed in the nervous system and is closely associated with neuronal survival and synapse formation. Elevated expression of *IL10RB* has been observed in the brains of Alzheimer's disease (AD) patients and mouse models (Luo et al. 2022), suggesting a potential role in AD pathogenesis. In the central nervous system, high levels of IL‐10 can suppress the expression and secretion of pro‐inflammatory cytokines (Frei et al. 1994; Peñaloza et al. 2016). In bats, such elevated IL‐10 expression may impair the ability to mount effective responses to infections. Overall, the immune profile of solitary males suggests chronic immune activation, potentially limiting their reproductive and competitive success compared to resident males.

### 4.2 Enrichment Analysis: Insights Into Ecological and Physiological Adaptations

KEGG pathway enrichment analysis revealed that the differentially expressed genes (DEGs) were primarily enriched in pathways including “Lysosome” and “Endocrine and other factor‐regulated calcium reabsorption”. Notably, all DEGs within these pathways were significantly upregulated in resident males. The lysosome pathway plays a crucial role in processes such as inflammation, antigen presentation, and the maintenance of long‐lived immune cells (Gros and Muller 2023), suggesting that resident males may have enhanced resistance to inflammation and greater immune longevity, potentially providing potential fitness advantages. Mammalian reproduction and development rely on the precise coordination of processes such as cell division, specification, migration, and apoptosis. These processes are regulated both spatially and temporally through chemical and mechanical signaling mechanisms (Webb and Miller 2003). As a secondary messenger, intracellular calcium signaling decodes and integrates signals from the extracellular environment, mediating interactions between chemical and physical cues (Berridge et al. 2000; Berridge et al. 1998). Therefore, calcium‐mediated signal transduction is fundamental to mammalian development and reproduction (Stewart and Davis 2019). The significant upregulation of genes within the calcium reabsorption pathway in resident males suggests that these bats may enhance their survival and fitness through the regulation of calcium signaling. Furthermore, these pathways are closely linked to metabolic regulation, cellular homeostasis, and signal transduction, reflecting adaptive differences in resource utilization and molecular mechanisms between resident and solitary males under sexual selection pressures.

Gene Ontology (GO) enrichment analysis revealed that DEGs in resident and solitary males were primarily enriched in key biological processes, including “protein binding”, “RNA binding” and “cytosol”, which are essential for cellular function. Additionally, significant enrichment was observed in terms related to immunity, cellular stress, and signal regulation, such as “positive regulation of innate immune response”, “response to hydrogen peroxide”, and “Rho protein signal transduction”. Our results revealed molecular differences in physiological pathways that may underlie behavioral divergence between resident and solitary males.

### 4.3 Limitations

We successfully assembled the transcriptome of *Tylonycteris pachypus* using RNA‐seq, providing valuable insights into its transcriptional landscape. Nevertheless, several limitations warrant consideration.

Although the reference assembly was of high quality, 25.8% of target genes remained incompletely recovered—a reflection of the persistent challenges in *de novo* transcriptome assembly for non‐model organisms. In addition, sampling difficulties and ethical constraints restricted our sample size; expanding the number of biological replicates would almost certainly improve assembly completeness and gene coverage. Furthermore, the relatively small number of differentially expressed genes (DEGs) limits our ability to draw definitive conclusions. Besides, reliance on a single tissue introduces potential bias due to tissue‐specific expression. Future work should integrate RNA‐seq data from multiple tissues to enable comparative analyses and to illuminate the evolutionary mechanisms underpinning sexual selection in *T. pachypus* .

## Author Contributions

**Chuyu Lv:** formal analysis (equal), visualization (equal), writing – original draft (lead). **Libiao Zhang:** resources (equal). **Ke Li:** validation (equal), writing – original draft (equal). **Panyu Hua:** methodology (equal), writing – review and editing (equal).

## Conflicts of Interest

The authors declare no conflicts of interest.

## Supporting information


**Appendix S1:** ece372116‐sup‐0001‐AppendixS1.docx.

## Data Availability

All raw data are available in the Dryad Digital Repository. Reviewers can access them via the following link: https://doi.org/10.5061/dryad.2547d7x2v.
