# Supplementary material for: Transcriptome Analyses Revealed the Genetic Advantages in Polygynous Males of Tylonycteris pachypus
Source: Ecol Evol. 2025 Sep 10;15(9):e72116. doi: 10.1002/ece3.72116 (PMC12422751; doi:10.1002/ece3.72116)
Supplement: Supplementary file 1 — Appendix S1: ece372116‐sup‐0001‐AppendixS1.docx. [file ECE3-15-e72116-s001.docx]

**Appendix 1: The main bioinformatics workflow of this study.**

**Appendix 2: Significantly Differentially Expressed Genes**

**Appendix 3: Significant Enrichment Results of Differentially Expressed Genes**

**The main bioinformatics workflow of this study**

**# 1. Quality control with Trimmomatic**

java -jar $TT/trimmomatic-0.36.jar PE -threads 30 -phred33 \

152598_R1_raw.fq 152598_R2_raw.fq \

152598_R1_qc.fq output_r1_unpaired.fq \

152598_R2_qc.fq output_r2_unpaired.fq \

ILLUMINACLIP:$TT/adapters/TruSeq2-PE.fa:2:30:10 \

LEADING:3 TRAILING:3 SLIDINGWINDOW:4:15 MINLEN:36

##The above operation is performed on the raw sequencing data of all individuals, using sample 152598 as an example.

**# 2. *De novo* assembly with Trinity > denovo_contigs**

Trinity --seqType fq --full_cleanup --max_memory 200G --left 152598_R1_qc.fq,152616_R1_qc.fq,152685_R1_qc.fq,152693_R1_qc.fq,152603_R1_qc.fq,152620_R1_qc.fq,152690_R1_qc.fq,152694_R1_qc.fq,152604_R1_qc.fq,152626_R1_qc.fq,152691_R1_qc.fq,152696_R1_qc.fq,152609_R1_qc.fq,152684_R1_qc.fq,152692_R1_qc.fq --right 152598_R2_qc.fq,152616_R2_qc.fq,152685_R2_qc.fq,152693_R2_qc.fq,152603_R2_qc.fq,152620_R2_qc.fq,152690_R2_qc.fq,152694_R2_qc.fq,152604_R2_qc.fq,152626_R2_qc.fq,152691_R2_qc.fq,152696_R2_qc.fq,152609_R2_qc.fq,152684_R2_qc.fq,152692_R2_qc.fq --CPU 36

**# 3. Remove redundancy with CD-Hit-EST**

nohup cd-hit-est -i trinity.fa -o denovo_conntigs -c 0.9 -G 0 -aS 0.9 -g 0 -M 0 -T 30

**# 4. Contigs assignation > annotated_denovo_contigs**

makeblastdb -in E.fuscus_rna.fna -dbtype nucl -out E.fuscus_rna_db

blastn -query denovo_contigs - E.fuscus_rna_db -out denovo_annotated_result -num_threads 30 -outfmt 6 -qcov_hsp_perc 70 -evalue 1e-5 -word_size 9 -perc_identity 70 -max_target_seqs 1 &

**# 5. Reads assignation**

cat E.fuscus_rna.fna annotated_denovo_contigs > merge.fna

makeblastdb -in merge.fna -dbtype nucl -out merge_db

cat *r1.fq *r2.fq > r12.fq &

nohup cat r12.fq | awk '{if(NR%4==1) {printf(">%s\n",substr($0,2));} else if(NR%4==2) print;}' > r12.fa &

blastn -query r2.fa -db r merge_db -out reads_assignation_results -num_threads 30 -outfmt 6 -evalue 1e-5

**# 6. Assembly for each contigs**

#6.1

#!/usr/bin/perl -w

use strict; use 5.016;

my $name = $ARGV[0];

my %hread;

open R1, "~/${name}_R1_qc.fq";

while (<R1>) {

chomp;

my $readidfull = $_;

my @line = split(/\s+/, $_);

my $id = substr($line[0], 1);

my $threeline = <R1>;

$threeline .= <R1>;

$threeline .= <R1>;

$hread{$id} = "$readidfull\n$threeline";

}

open R2, "~/${name}_R2_qc.fq";

while (<R2>) {

chomp;

my $readidfull = $_;

my @line = split(/\s+/, $_);

my $id = substr($line[0], 1);

my $threeline = <R2>;

$threeline .= <R2>;

$threeline .= <R2>;

$hread{$id} .= "$readidfull\n$threeline";

}

my %hashgeneid;

open BLAST, "./${name}_reads_assignation_results";

while (<BLAST>) {

chomp;

my @line = split(/\s+/, $_);

$hashgeneid{$line[1]}{$line[0]} = $hread{$line[0]};

}

open STDOUT, '>', "${name}_r1r2.fastq";

for my $key1 (keys %hashgeneid) {

print "##$key1\n";

my $hash2 = $hashgeneid{$key1};

for my $key2 (keys %$hash2) {

print $hashgeneid{$key1}{$key2};

}

}

#6.2

#!/usr/bin/bash

touch workbegin

mkdir -p contigs_collected

fadone="path_to_done_directory"

for fq in $(ls r1dir/*fastq.gz | cut -f 2 -d / | cut -f 1-2 -d _)

do

if [ ! -e contigs_collected/${fq}.fa.gz ]; then

spades.py -1 r1dir/${fq}_r1.fastq.gz -2 r2dir/${fq}_r2.fastq.gz -o ${fq}.dir \

-t 5 --cov-cutoff=auto --careful --rna

if [ -e ${fq}.dir/transcripts.fasta ]; then

cat ${fq}.dir/transcripts.fasta | gzip > ${fadone}/${fq}.fa.gz

fi

if [ -e ${fq}.dir/scaffolds.fasta ]; then

cp ${fq}.dir/scaffolds.fasta contigs_collected/${fq}.fa

rm r1dir/${fq}_r1.fastq.gz r2dir/${fq}_r2.fastq.gz

else

mv r1dir/${fq}_r1.fastq.gz failed_r1/

mv r2dir/${fq}_r2.fastq.gz failed_r2/

fi

rm -r ${fq}.dir &

gzip contigs_collected/${fq}.fa

fi

done

wait

touch workfinished

#6.3

#!/bin/bash

makeblastdb -in E.fuscus_rna.fna -dbtype nucl -out E.fuscus_rna_db

for gene_file in *.fasta; do

gene_id=$(basename "$gene_file" .fasta)

blastn -query "$gene_file" -db E.fuscus_rna_db -outfmt 6 -evalue 1e-5 -out blast_results_"$gene_id".txt

longest_contig=""

longest_length=0

while read -r line; do

contig_id=$(echo $line | awk '{print $2}')

alignment_length=$(echo $line | awk '{print $3}')

if [ $alignment_length -gt $longest_length ]; then

longest_length=$alignment_length

longest_contig=$contig_id

fi

done < blast_results_"$gene_id".txt

echo "$gene_id $longest_contig" >> transcript-guided_contigs.txt

done

> selected_longest_contigs.fasta

while read -r line; do

gene_id=$(echo $line | awk '{print $1}')

contig_id=$(echo $line | awk '{print $2}')

gene_file="${gene_id}.fasta"

seqtk subseq "$gene_file" <(echo ">${contig_id}") >> selected_longest_contigs.fasta

done < transcript-guided_contigs

**#7. Construction of reference transcriptome of *Tylonycteris pachypus***

#!/bin/bash

cut -f 2 denovo_annotated_result | sort | uniq > annotated_contigs.txt

> denovo_contigs_filtered.fa

awk '/^>/ {contig_id = substr($1, 2)} {if (!seen[contig_id] && contig_id != "") {seen[contig_id]=1; print_header=1}}

!/^>/ {if (print_header) {print $0}}' denovo_contigs.fa > denovo_contigs_filtered.fa

mv denovo_contigs_filtered.fa denovo_contigs

cat denovo_contigs transcript-guided_contigs > reference_transcriptome_of_T.pachypus.fa

**#8. Quantitative analysis by Bowtie2 and RSEM.**

rsem-prepare-reference --transcript-to-gene-map gene_trans_map --bowtie2 reference_transcriptome_of_T.pachypus.fa index -p 30

command="rsem-calculate-expression --no-bam-output --bowtie2 --paired-end $file ${file/_R1_/_R2_} index $output_dir -p 30 &"

for file in *.fq.gz; do

gunzip -c "$file" > /path/to/ "${file%.gz}";

done &

**#9. Perform differential expression analysis using DESeq2.**

library(tximport)

library(DESeq2)

files <- c("152598.genes.results", "152603.genes.results", "152604.genes.results", "152609.genes.results", "152692.genes.results", "152620.genes.results", "152626.genes.results", "152684.genes.results", "152690.genes.results", "152694.genes.results")

names(files) <- c("152598", "152603", "152604", "152609", "152692", "152620", "152626", "152684", "152690", "152694")

txi.rsem <- tximport(files, type = "rsem", txIn = FALSE, txOut = FALSE)

head(txi.rsem$counts)

sampleTable <- data.frame(

condition = factor(c("control","control","control","control","control","treatment","treatment","treatment","treatment","treatment"))

)

rownames(sampleTable) <- colnames(txi.rsem$counts)

rounded_counts <- round(txi.rsem$counts)

dds <- DESeqDataSetFromMatrix(countData = rounded_counts,

colData = sampleTable,

design = ~ condition)

dds <- DESeq(dds)

res <- results(dds)

summary(res)

sig_genes <- res[which(res$padj < 0.05), ]

nrow(sig_genes)

**Significantly Differentially Expressed Genes**

| Gene Name | log2FoldChange | Corrected *P*-Value |
| --- | --- | --- |
| Egl-9 family hypoxia inducible factor 2(*EGLN2*) | 22.445 | <0.001 |
| Coactosin like F-actin binding protein 1(*COTL1*) | -1.725 | 0.025 |
| Tec protein tyrosine kinase(*TEC*) | 6.098 | <0.001 |
| Solute carrier family 2 member 3(*SLC2A3*) | -24.607 | <0.001 |
| RNA binding motif protein 27(*RBM27*) | 4.597 | 0.033 |
| N-terminal asparagine amidase(*NTAN1*) | 1.919 | 0.023 |
| Nuclear transcription factor, X-box binding 1(*NFX1*) | 6.893 | 0.036 |
| Zinc finger matrin-type 5(*ZMAT5*) | -1.303 | 0.021 |
| Fem-1 homolog C(*FEM1C*) | -5.887 | 0.002 |
| Replication protein A1(*RPA1*) | 0.717 | 0.033 |
| RIO kinase 3(*RIOK3*) | 4.413 | 0.021 |
| Clathrin heavy chain(*CLTC*) | 9.921 | 0.021 |
| SCAN domain containing 1(*SCAND1*) | 3.290 | 0.008 |
| Signal peptide peptidase like 2B(*SPPL2B*) | 0.848 | 0.046 |
| VANGL planar cell polarity protein 2(*VANGL2*) | -1.122 | 0.019 |
| Methyltransferase 14, N6-adenosine-methyltransferase non-catalytic subunit(*METTL14*) | 6.543 | 0.027 |
| RB binding protein 7, chromatin remodeling factor(*RBBP7*) | -0.856 | 0.025 |
| Synapse defective Rho GTPase homolog 1(*SYDE1*) | 6.389 | 0.015 |
| Calreticulin(*CALR*) | 0.431 | 0.020 |
| Rab geranylgeranyltransferase subunit alpha(*RABGGTA*) | 29.052 | <0.001 |
| High mobility group box 3(*HMGB3*) | -1.778 | <0.001 |
| Splicing factor 1(*SF1*) | 6.378 | 0.033 |
| ATPase H+ transporting accessory protein 1(*ATP6AP1*) | 22.637 | <0.001 |
| Ribophorin I(*RPN1*) | 7.912 | 0.007 |
| Urocanate hydratase 1(*UROC1*) | 4.552 | 0.021 |
| Nucleoporin 58(*NUP58*) | 1.525 | 0.045 |
| Transmembrane protein 108(*TMEM108*) | -1.397 | 0.024 |
| Von Willebrand factor A domain containing 3B(*VWA3B*) | -6.530 | <0.001 |
| N-alpha-acetyltransferase 40, NatD catalytic subunit(*NAA40*) | -1.141 | <0.001 |
| C-X9-C motif containing 4(*CMC4*) | -1.407 | 0.037 |
| Ubiquitin conjugating enzyme E2 S(*UBE2S*) | -0.697 | 0.035 |
| Glutamate rich 3(*ERICH3*) | 0.615 | 0.019 |
| Nexilin F-actin binding protein(*NEXN*) | -6.103 | 0.023 |
| Vacuolar protein sorting 4 homolog A(*VPS4A*) | -0.289 | 0.009 |
| Riboflavin kinase(*RFK*) | -8.921 | 0.008 |
| Nuclear transcription factor, X-box binding 1(*NFX1*) | 6.893 | 0.036 |
| Butyrylcholinesterase(*BCHE*) | 0.437 | 0.040 |
| 2-5-oligoadenylate synthetase like(*OASL*) | -1.312 | 0.009 |
| Zinc finger protein 451(*ZNF451*) | -1.593 | 0.029 |
| LysM domain containing 2(*LYSMD2*) | -0.410 | 0.040 |
| CUGBP Elav-like family member 1(*CELF1*) | 1.146 | 0.046 |
| Mitochondrial rRNA methyltransferase 3(*MRM3*) | -5.468 | 0.004 |
| Protein phosphatase 1 regulatory subunit 8(*PPP1R8*) | 2.094 | 0.000 |
| Methyl-CpG binding domain protein 1(*MBD1*) | 0.535 | 0.037 |
| RIO kinase 3(*RIOK3*) | 4.413 | 0.021 |
| Clathrin heavy chain(*CLTC*) | 9.921 | 0.021 |
| Optineurin(*OPTN*) | 23.966 | <0.001 |
| Cytochrome c1, heme protein, mitochondrial(*LOC103302658*) | 23.409 | <0.001 |
| Solute carrier family 12 member 8(*SLC12A8*) | 1.267 | 0.013 |
| Cadherin 13(*CDH13*) | -23.123 | 0.000 |
| Ryanodine receptor 1(*RYR1*) | 0.915 | 0.041 |
| SH3 and multiple ankyrin repeat domains 1(*SHANK1*) | 25.922 | <0.001 |
| Egl-9 family hypoxia inducible factor 2(*EGLN2*) | 22.445 | <0.001 |
| Egl-9 family hypoxia inducible factor 2(*EGLN2*) | 22.445 | <0.001 |
| Patr class I histocompatibility antigen, A-126 alpha chain-like(*LOC129147758*) | 7.721 | 0.024 |
| Ankyrin repeat domain containing 11(*ANKRD11*) | 23.300 | <0.001 |
| Ankyrin repeat domain containing 11(*ANKRD11*) | 23.300 | <0.001 |
| Coiled-coil domain containing 190(*CCDC190*) | -5.740 | 0.029 |
| RAS protein activator like 2(*RASAL2*) | -7.156 | <0.001 |
| Choline/ethanolamine phosphotransferase 1(*CEPT1*) | 22.909 | <0.001 |
| Ubiquitin protein ligase E3A(*UBE3A*) | 1.841 | 0.024 |
| Interleukin 10 receptor subunit beta(*IL10RB*) | -1.153 | 0.050 |
| Salt inducible kinase 1(*SIK1*) | -22.939 | <0.001 |
| Zinc finger C4H2-type containing(*ZC4H2*) | 10.417 | <0.001 |
| N-terminal asparagine amidase(*NTAN1*) | 1.919 | 0.023 |
| Intraflagellar transport 22(*IFT22*) | 0.470 | 0.004 |
| Semaphorin 6A(*SEMA6A*) | 6.307 | 0.037 |
| Adaptor related protein complex 3 subunit sigma 1(*AP3S1*) | 2.108 | 0.047 |
| GTP dependent ribosome recycling factor mitochondrial 2(*GFM2*) | -1.080 | 0.036 |
| Microtubule associated serine/threonine kinase family member 4(*MAST4*) | 1.273 | 0.025 |
| Mitogen-activated protein kinase kinase kinase 9(*MAP3K9*) | 22.251 | <0.001 |
| Microtubule affinity regulating kinase 3(*MARK3*) | -8.579 | <0.001 |
| C2 calcium dependent domain containing 4A(*C2CD4A*) | -2.335 | 0.005 |
| MINDY lysine 48 deubiquitinase 3(*MINDY3)* | 8.133 | <0.001 |
| Mastermind like domain containing 1(*MAMLD1*) | -8.474 | <0.001 |
| Chloride voltage-gated channel 3(*CLCN3*) | 9.599 | 0.025 |
| Solute carrier family 25 member 48(*SLC25A48*) | 0.785 | 0.033 |
| Thioredoxin, mitochondrial-like(*LOC103298793*) | 1.383 | <0.001 |
| Mannose-6-phosphate receptor, cation dependent(*M6PR*) | 22.994 | <0.001 |
| Ribosomal modification protein rimK like family member B(*RIMKLB*) | 0.483 | 0.031 |
| GPALPP motifs containing 1(*GPALPP1*) | 0.871 | 0.026 |
| Plasma kallikrein-like(*LOC103290370*) | 4.791 | 0.021 |
| FGR proto-oncogene, Src family tyrosine kinase(*FGR*) | -6.434 | 0.032 |
| Zinc finger protein 711(*ZNF711*) | 0.688 | 0.040 |
| Protein kinase cAMP-activated catalytic subunit beta(*PRKACB*) | 0.868 | 0.001 |
| Uronyl 2-sulfotransferase(*UST*) | 8.990 | 0.005 |
| PNN interacting serine and arginine rich protein(*PNISR*) | 6.821 | 0.004 |
| R3H domain containing 1(*R3HDM1*) | -0.888 | 0.023 |
| Heat shock protein family D (Hsp60) member 1(*HSPD1*) | 1.965 | 0.001 |
| Aldehyde dehydrogenase 1 family member L1(*ALDH1L1*) | 0.954 | 0.037 |
| Inhibitor of growth protein 1-like(*LOC103285855*) | 5.063 | 0.001 |
| RNA binding motif protein 7(*RBM7*) | 7.770 | <0.001 |
| FERM domain containing 8(*FRMD8*) | -1.799 | 0.004 |
| COPI coat complex subunit beta 1(*COPB1*) | -23.903 | <0.001 |
| Lysine demethylase 2A(*KDM2A*) | 1.839 | 0.015 |
| Cofilin 1(*CFL1*) | 10.385 | <0.001 |
| Splicing factor 1(*SF1*) | 6.378 | 0.033 |
| Nucleotide binding oligomerization domain containing 1(*NOD1*) | -1.372 | 0.037 |
| Tax1 binding protein 1(*TAX1BP1*) | -5.989 | 0.040 |
| Heterogeneous nuclear ribonucleoprotein A2/B1(*HNRNPA2B1*) | 1.578 | 0.035 |
| Spastin(*SPAST*) | 22.244 | <0.001 |
| Prolyl endopeptidase like(*PREPL*) | 24.960 | <0.001 |
| Zinc finger MIZ-type containing 1(*ZMIZ1*) | 8.856 | 0.033 |
| ATPase Na+/K+ transporting subunit beta 3(*ATP1B3*) | 24.087 | <0.001 |
| Shisa family member 5(*SHISA5*) | -1.998 | 0.024 |
| Coiled-coil domain containing 51(*CCDC51*) | -1.781 | 0.010 |
| Leucyl-tRNA synthetase 2, mitochondrial(*LARS2*) | 8.147 | <0.001 |
| Small nucleolar RNA SNORD37(*LOC114234874*) | 7.237 | <0.001 |
| Uncharacterized LOC129147137(*LOC129147137*) | 0.731 | 0.029 |
| Platelet activating factor acetylhydrolase 1b regulatory subunit 1(*PAFAH1B1*) | 22.262 | 0.000 |
| Uncharacterized LOC129147302(*LOC129147302*) | 0.647 | 0.038 |
| Potassium channel tetramerization domain containing 12(*KCTD12*) | 3.812 | 0.050 |
| Uncharacterized LOC129151028(*LOC129151028*) | 22.818 | <0.001 |
| Small nucleolar RNA SNORA54(*LOC129151533*) | 2.500 | 0.019 |
| BMP2 inducible kinase(*BMP2K*) | 1.517 | 0.029 |

**Significant Enrichment Results of Differentially Expressed Genes**

| Database | Term | ID | Gene Counts | Corrected *P*-Value |
| --- | --- | --- | --- | --- |
| KEGG | Lysosome | hsa04142 | 4 | 0.014 |
|  | Endocrine and other factor-regulated calcium reabsorption | hsa04961 | 3 | 0.015 |
|  | Human papillomavirus infection | hsa05165 | 5 | 0.040 |
| GO | Protein binding | GO:0005515 | 70 | <0.001 |
|  | Cytosol | GO:0005829 | 35 | <0.001 |
|  | ATP binding | GO:0005524 | 18 | <0.001 |
|  | Nucleoplasm | GO:0005654 | 27 | <0.001 |
|  | Nucleus | GO:0005634 | 33 | <0.001 |
|  | Extracellular exosome | GO:0070062 | 20 | <0.001 |
|  | RNA binding | GO:0003723 | 16 | <0.001 |
|  | Cytoplasm | GO:0005737 | 27 | 0.002 |
|  | Axon cytoplasm | GO:1904115 | 4 | 0.002 |
|  | Nuclear membrane | GO:0031965 | 6 | 0.004 |
|  | Response to cocaine | GO:0042220 | 3 | 0.012 |
|  | Plasma membrane | GO:0005886 | 25 | 0.012 |
|  | Single-stranded telomeric DNA binding | GO:0043047 | 2 | 0.012 |
|  | Protein phosphorylation | GO:0006468 | 7 | 0.012 |
|  | Viral process | GO:0016032 | 7 | 0.012 |
|  | MRNA binding | GO:0003729 | 5 | 0.012 |
|  | Positive regulation of xenophagy | GO:1904417 | 2 | 0.012 |
|  | Neuromuscular process controlling balance | GO:0050885 | 3 | 0.014 |
|  | Endosome membrane | GO:0010008 | 5 | 0.014 |
|  | Unmethylated CpG binding | GO:0045322 | 2 | 0.014 |
|  | Nuclear envelope reassembly | GO:0031468 | 2 | 0.014 |
|  | Response to hydrogen peroxide | GO:0042542 | 3 | 0.014 |
|  | Centrosome | GO:0005813 | 7 | 0.014 |
|  | Perinuclear region of cytoplasm | GO:0048471 | 8 | 0.017 |
|  | G-rich strand telomeric DNA binding | GO:0098505 | 2 | 0.017 |
|  | Cellular response to brain-derived neurotrophic factor stimulus | GO:1990416 | 2 | 0.017 |
|  | Rho protein signal transduction | GO:0007266 | 3 | 0.017 |
|  | Apoptotic process | GO:0006915 | 7 | 0.017 |
|  | Exit from mitosis | GO:0010458 | 2 | 0.017 |
|  | Retrograde axonal transport | GO:0008090 | 2 | 0.017 |
|  | Pre-mRNA intronic binding | GO:0097157 | 2 | 0.017 |
|  | Isomerase activity | GO:0016853 | 2 | 0.025 |
|  | mRNA processing | GO:0006397 | 4 | 0.025 |
|  | Receptor-mediated endocytosis | GO:0006898 | 4 | 0.027 |
|  | Double-stranded RNA binding | GO:0003725 | 3 | 0.028 |
|  | Endosome | GO:0005768 | 5 | 0.028 |
|  | Golgi apparatus | GO:0005794 | 9 | 0.028 |
|  | Zinc ion binding | GO:0008270 | 8 | 0.031 |
|  | Protein targeting to lysosome | GO:0006622 | 2 | 0.033 |
|  | Cellular protein modification process | GO:0006464 | 3 | 0.034 |
|  | Androgen receptor signaling pathway | GO:0030521 | 2 | 0.034 |
|  | Brain morphogenesis | GO:0048854 | 2 | 0.040 |
|  | Cytoskeleton-dependent cytokinesis | GO:0061640 | 2 | 0.042 |
|  | Transport vesicle | GO:0030133 | 3 | 0.044 |
|  | Membrane | GO:0016020 | 13 | 0.046 |
|  | Protein-containing complex binding | GO:0044877 | 5 | 0.046 |
|  | Positive regulation of innate immune response | GO:0045089 | 2 | 0.048 |
